# Exploring the hub mechanisms of ischemic stroke based on protein-protein interaction networks related to ischemic stroke and inflammatory bowel disease

**DOI:** 10.1038/s41598-023-27459-w

**Published:** 2023-01-31

**Authors:** Wei Hu, Ping Li, Nianju Zeng, Sheng Tan

**Affiliations:** 1 Department of Neurology, Zhujiang Hospital, Southern Medical University, Guangzhou, 510280 China; 2 Department of Rehabilitation, Xiangya Bo’ai Rehabilitation Hospital, Changsha, 410004 China

**Keywords:** Genetics, Immunology, Neuroscience, Neurology

## Abstract

Ischemic stroke is highly concerning because it often leads to severe long-term neurological disability. Among clinical trials, ischemic stroke and inflammatory bowel disease interactions have been increasingly reported in recent years. Therefore, using bioinformatics approaches to explore novel protein interactions between them is of interest. We performed this exploratory analysis by using bioinformatics tools such as string to analyze gene data downloaded from NHGRI-GWAS data related to ischemic stroke and inflammatory bowel disease. We constructed a prospective protein interaction network for ischemic stroke and inflammatory bowel disease, identifying cytokine and interleukin-related signaling pathways, Spliceosome, Ubiquitin–Proteasome System (UPS), Thrombus, and Anticoagulation pathways as the crucial biological mechanisms of the network. Furthermore, we also used data-independent acquisition mass spectrometry (DIA-MS) to detect differential protein expression in eight samples, which also suggested that immune system, signal transduction, and hemostasis-related pathways are key signaling pathways. These findings may provide a basis for understanding the interaction between these two states and exploring possible molecular and therapeutic studies in the future.

## Introduction

Ischemic stroke is the most common type of cerebrovascular disease, with four high characteristics such as high morbidity, high disability, high recurrence and high mortality, which brings a heavy medical and economic burden to patients' families and society. By 2030, almost 3.9% of the adult population in the United States would have had a stroke, considering that there has been a 20.5% increase in its prevalence since 2012^1^. Of all strokes, 87% are ischemic strokes (ISs)^2^. In the United States, strokes have caused serious and long-term neurological disability in^3^ approximately 3% of the males and 2% of the females that have suffered them. It is predicted that the total direct medical costs of stroke-related diseases will increase between 2015 and 2035 from $36.7 billion to $94.3 billion, with much of the anticipated increase arising from affliction of people more than 80 years of age^4^. The Global Burden of Disease Study 2019 (GBD 2019) showed that the global prevalence of stroke was 104.2 million and that of IS was 82.4 million^5^.

Considering the high rates of morbidity and mortality, ferreting out the causes and the potential molecular mechanisms of stroke and identifying molecular biomarkers for early diagnosis, preventive treatment, and precise therapy are critically important. Familial epidemiological studies suggest that stroke has a genetic component. Genes modestly associated with stroke have been identified in several studies. Several heritable disorders associated with stroke have also been reported^6^.

Inflammatory bowel disease (IBD), including ulcerative colitis (UC) and Crohn's disease (CD). It was first identified in the western developed countries, and the cause is still unknown, but the number of patients in Europe and the United States has accounted for 0.5% of the world population. It is an idiopathic inflammatory disease of the intestinal tract involving the ileum, rectum and colon. Clinical manifestations include diarrhea, abdominal pain, and even bloody stools. In the past 20 years, the incidence and prevalence of inflammatory bowel disease (IBD) has risen steeply in developing countries in Asia, South America, the Middle East, and Africa, with the rate of increase particularly significant in South America and East Asia, and has become a global disease A 2017 retrospective study of the incidence and prevalence of IBD worldwide published in The Lancet^2^ showed that the incidence in Europe and the United States has reached a plateau while non-Western countries are in the first phase of new case growth. More than 2 million Europeans and 1.5 million North Americans currently suffer from inflammatory bowel disease. And according to 2014 data from the Chinese Center for Disease Control and Prevention, the total number of IBD cases in China between 2005 and 2014 was about 350,000. One study estimated that by 2025, the number of people with IBD in China will reach 1.5 million^7^.

Compared with the general population, there was a significant difference in the risk of stroke in patients with IBD^8^. Patients with IBD have an abnormal immune response to microbial dysbiosis, leading to a sustained stimulated pro-inflammatory state, and pro-inflammatory Th1, Th17, and γδ T cells are often associated with increased inflammatory injury, while subsets of T cells can help or exacerbate ischemic brain injury^9,10^. In addition, inflammatory bowel disease increases patients' risk of atrial fibrillation^11^, predisposes patients to recurrent ischemic cerebrovascular events, and stroke survivors with inflammatory bowel disease are more likely to have a worse prognosis than those without inflammatory bowel disease^12^. In addition, one study used two-sample bidirectional Mendelian randomization (MR) to analyze the relationship between stroke and osteoarthritis (OA), a common general inflammatory disease. Among the three categories of OA at any site, knee OA and hip OA, hip OA was found to be a potential risk factor for overall stroke, IS and small vessel IS. The mechanisms linking this site-specific OA to stroke remain to be further studied^13^. Potential mechanisms between inflammatory bowel disease and ischemic stroke may include activation of the innate immune system, systemic inflammation leading to vascular endothelial dysfunction, increased cholesterol biosynthesis, and thrombotic states. All of these may exacerbate atherosclerotic lesions and increase the risk of atherosclerosis, thereby promoting cerebrovascular events, including large- and small-vessel cerebral infarcts. To this end, protein interaction data obtained from a wide range of cellular and biochemical model systems can be used to build protein–protein interaction (PPI) networks from genes associated with inflammatory bowel disease and stroke, which would enable a better understanding of genetics for ischemic stroke pathogenesis in terms of biological pathway and functional analysis, integrating relevant genetic risk factors. As biotechnology continues to advance, PPI network analysis may be a powerful time- and cost-efficient approach to identify potential biological pathways in ischemic stroke, key players or candidate genes involved in the inflammatory bowel disease-stroke linkage.

Therefore, the aim of this study was to identify key proteins and bioregulatory pathways involved in the physiopathology of inflammatory bowel disease and stroke through bioinformatic analysis and application of surveillance-related proteins.

## Materials and methods

### Data collection

We downloaded data on inflammatory bowel disease and ischemic stroke from the NHGRI-GWAS database by entering the keywords ischemic stroke and inflammatory bowel disease, respectively, in the search field of the NHGRI-GWAS database (https://www.ebi.ac.uk/gwas/, accessed on 27 January 2022). The GWAS-related studies on ischemic stroke included more than 2,951,164 individuals covering Europeans, East Asians, South Asians, African Americans or African Caribbean, Hispanics or Latinos. The GWAS-related studies on inflammatory bowel disease included approximately 640,515 individuals from sub-Saharan African, European, East Asian, South Asian, African American or African Caribbean, Hispanic, or Latino ethnic populations. The GWAS data were all from different populations because none of the included studies combined data from both ischemic stroke and inflammatory bowel disease conditions.

### Data organization and analysis

We used Microsoft Office Excel to perform basic processing of the data. After removing duplicates by excel, the total number of genes involved in ischemic stroke was 418, while the number involved in inflammatory bowel disease was 400.We then accessed the string online website (version 11.5) and selected the multi-protein model to construct the protein–protein interaction network. We set the maximum number of interactors displayed to 10 and the minimum required interaction score of 0.4 for the first analysis. Based on the analysis, we then determined if a higher interaction score of 0.7 or 0.9 would be chosen as the cutoff score for the analysis.

### Construction and analysis of protein–protein interaction network

The STRINGdb v11.5 package (https://string-db.org/cgi/input.pl?) was used toconstruct a PPI network of the proteins encoded by the genes. Furthermore, Cytoscape v3.7.1 was utilized to construct a PPI network, considering confidence a score ≥ 0.9 and maximum number of interactors = 10 as cut-off criteria, and the interaction of the candidate gene-encoded proteins was analyzed. Moreover, the Network Analyzer plug-in was used to calculate node degrees, i.e., the number of interconnections useful to filter hub genes from the PPI network. The Molecular Complex Detection (MCODE) plug-in of the Cytoscape tool was employed to visualize the significant gene clusters in IS with a degree cutoff = 2, node score cutoff = 0.2, k-core = 2, and max. depth = 100. The criteria for selecting the two most significant clusters were set as MCODE scores ≥ 4 and number of nodes ≥ 4. The corresponding proteins in the central nodes might be key proteins and hub candidate genes with important physiological regulatory functions.

### GO and biological pathway enrichment analysis

GO and pathway enrichment analyses of genes were performed using multiple online databases. We submitted the genes to the DAVID online program (https://david.ncifcrf.gov/; version: 6.8) with p < 0.05 as the cut-off criterion. Besides, GO and pathway analyses were carried out using KEGG (http://www.genome.jp/kegg), Reactome (http://www.reactome.org), BioCyc (http://biocyc.org), and PANTHER (http://www.pantherdb.org)^14^. The enriched GO terms were ranked according to their p-values and visualized as bar charts; p < 0.05 was considered as statistically significant^15,16^.

### Corroboration of key pathways

To further validate the key signaling pathways obtained from the above bioinformatic analysis, we used data-independent acquisition mass spectrometry (DIA-MS), one of the highly regarded mass spectrometry acquisition techniques in recent years, to identify and quantify proteins in samples from 8 blood samples (4 ischemic stroke samples and 4 normal population samples). The main steps were as follows: first, whole blood was collected using a vacuum blood collection tube and gently mixed by inverting up and down 5–6 times; the blood was left to stand for 30 min at 4 °C to allow coagulation; the supernatant was immediately taken by centrifugation at 1700*g* for 10 min at 4 °C; the upper layer of yellowish serum was transferred to a centrifuge tube using a pipette; liquid nitrogen was snap frozen for 5 min and stored at − 80 °C for backup. Then, protein extraction was performed, and then a portion of the prepared total protein solution was trypsinized, and the enzymatically cleaved peptides were desalted using a SOLA™ SPE 96-well plate for LC–MS/MS high-resolution mass spectrometry detection (Full MS: Resolution = 120,000, AGC target = 3e6, Maximum injection time = 50 ms, Scan range = 350–1250 m/z; MS2: Resolution = 15,000, AGC target = 1e5, Maximum injection time = 35 ms, Scan range = 200–2000 m/z, Isolation window = 1.4 m/z, Normalized Collision Energy = 28), and the high-abundance proteins were removed using a MilliPore de-abundance kit before detection. The LC–MS/MS raw files were imported into Spectronaut Pulsar software for matching, and the machine signals were transformed into peptide and protein sequence information, and then combined with sequence information, peptide retention time, and fragment ion information for the spectral library building to facilitate the subsequent DIA analysis. Based on obtaining plausible proteins, we adopted the following criteria to screen the differential proteins: FoldChange ≥ 1.2, P ≤ 0.05 as up-regulated proteins; FoldChange ≤ 0.833, P ≤ 0.05 as down-regulated proteins. To further understand the biological functional information of differential proteins, we performed GO and pathway enrichment analysis on candidate DEPs using multiple online databases.

## Results

### Identification of genes in ischemic strokes and inflammatory bowel disease

NHGRI-GWASCatalog supplies a publicly hand-curated collection of published GWAS assaying more than 100,000 single-nucleotide polymorphisms (SNPs) and all SNP-trait associations with p < 1 × 10–5.The Catalog includes 5595 curated publications of 195,610 SNPs and 326,947 associations. In addition to SNP-trait association data, Catalo is a global public source that archives and makes freely available high-throughput functional genomics data submitted by researchers. The GWAS datasets of ischemic stroke (EFO ID: HP_0002140, include 17 reported traits with 314 Associations) and inflammatory bowel disease (EFO ID: EFO_0003767, include 11 reported traits with 526 Associations, excluding child trait data) were taken from this repository, we extracted 418, and 400 genes. After performing an integrated bioinformatical analysis using jvenn (https://www.vandepeerlab.org/?q=tools/venn-diagrams)^17^, the intersection of these datasets yielded 12 genes, and the total number of unique elements is 806 (Table 1).

**Table 1:** Identification of genes in ischemic strokes and inflammatory bowel disease (calculate and draw custom Venn diagrams).

List names	Number of genes	Number of unique elements
Inflammatory bowel disease	400	400
Ischemic stroke	418	418
Overall number of unique elements		806


Names	Total	Genes
Inflammatory Bowel Disease Ischemic Stroke	12	DELEC1 ERAP2 SOX5 ADGRF1 PHACTR2 ATP6V1G3 SH2B3 F5 Y_RNA ATXN2 PTCH1 PTPRC
Ischemic Stroke	406	RPTOR LINC01013 KCNMA1 DEFA3 KIF26B NDUFS6 LINC01435 INPP5F COX7A2L ACOT6 FOXP4 GLTP DCHS2 COL4A2 PRDM8 RNU4-64P LINC01893 TECTA MMP7 MC4R R3HCC1L BRWD1P2 HNRNPA1P67 CGNL1 CENPQ CHORDC1 CRYBB2P1 RCN1P1 MCPH1-AS1 PLCB2 STN1 TSPAN15 LATS2 SIGLEC15 TMOD4 KCNK3 GDA HTR4 PRKCZ STARP1 UNQ6494 VWDE MTND1P24 PTPRD RORA NSF GPR37 NFIA CDH13 PTPRN2 TANGO2 LINC02125 ARHGAP21 MIR4277 RPL18P1 RAB19 LINC00923 PLCB4 UBE2V1P16 SLC44A2 MAPKAPK5-AS1 F11-AS1 TERF2IP DSCAML1 A1CF FGGY Metazoa_SRP FAM170B-AS1 LINC01915 SRRM4 USP46 CCDC39 NDUFB10P1 RPS12P23 LINC02267 DAB1 RPL17P35 SLC22A7 GALNT13 ESRRAP2 SGO1P2 COL28A1 LINC02884 CHMP4AP1 NCAM2 VAT1L ALDH2 TRAPPC11 SWAP70 RNU6-560P FGA SLC1A1 PRTFDC1 SEMA3A ULK4 CYSLTR2 WIZ FGG TPT1P9 HELLPAR PMF1 SMARCA4 RNF17 RBFOX1 LINC00924 TTC8 PCDH7 QRICH2 LINC02400 OPCML RNA5SP430 SECISBP2L RN7SL474P AMBN CD36 PRPF8 ENPEP MTND4P33 VN2R10P ELAPOR2 EPG5 HSD17B11 TRAV2 CCL16 SLC4A1AP APOD LINC01438 DNAH11 ADGRL3 KHDRBS3 NLRP5 GRK3 CDKN2B-AS1 ABO COL14A1 OSBPL1A CHD3 OR10J6P RN7SL73P ATPSCKMT RNU7-92P PTPRG GRIK2 TMEM273 MORN1 MIR8084 TNFRSF21 HTR3A ZNF474-AS1 CYP1B1-AS1 OR52B6 DEPDC1-AS1 FGB CCDC83 WNT2B CASZ1 FOXF2-DT B4GALT4-AS1 LINC01765 PPIAP22 PDZRN4 DNM2 OR4E1 HPS4 SDHAP3 C8orf86 PICALM ENAM BCL9L SLC36A2 TMEM74 RUNX1 TMEM170B ZBED1P1 RB1 ALDH1A2 FGF5 NAALADL2 RNU6-102P CXCL8 HNRNPA1P53 POP1 HDAC9 NALCN-AS1 GDI2 LINC01394 KNG1 LINC00640 C17orf102 SORBS2 SH3PXD2A TSPEAR LINC02426 RNU7-165P NDUFB8P1 ZNF626 GOSR2 ADAMTS13 TMEM243 PDE9A TP53TG1 COL4A1 ADAMTS3 GRM7 DNAH8 HTR3B ILF2P2 PITX2 TMEM123 LINC02511 FAF1 CST13P CDK6 SNTG2 SDR16C6P FZD7 LRCH1 RP1 RNU6-380P CADPS2 SRGAP3 FURIN TGFBR3 TMEM14A ANK2 CDKN1A ASTN2 LINC02709 ZNF516 SSPN KCTD13 USP36 MYRIP PRKCZ-AS1 XKR9 PATJ LINC01377 RHAG NTN4 NRCAM DISC1FP1 LINC02882 NYNRIN LINC02537 CDKN2C TTBK1 RN7SL363P EIF4A2 CST8 ICE2P2 COL6A3 LINC00545 STARD3NL ASPHD2 BOLA3P1 LINC00398 SDR16C5 AGMO MIX23P2 CRTAC1 F2 TMEM126B RNA5SP107 ZFHX3 TRAM2-AS1 ZNF77 PLRG1 LMX1B RASAL3 CNTNAP4 IRX1 ABCC1 RNU4ATAC9P DPPA3 SYCE1L VWF DEFA11P VPS33B ATP5MC1P8 PIK3C2G ALKBH8 RNU6-694P NPY1R RPL21P41 PSMB3 DTWD1 SLCO1B1 KCNK1 SFRP4 DDR2 LHFPL3 NRBF2P1 MPRIP ZFAND2A PPP2R5E TMEM132D RNU6-440P RUFY4 AACSP1 CDC5L LMNA PTPRT PDE3A MKRN1 SLC26A11 LYZL6 C10orf143 POU2F1 FUNDC2 GCATP1 LINC01522 TBC1D3P2 LAMA1 GALNT1 TRMT112P7 CDH11 MARCHF1 CPNE8-AS1 LRAT FAM95C MACROD2 MTX2 SLC14A1 RNU6-144P LINC02399 UAP1 USP28 CRISP2 ILF3 LINC01894 AGBL1 ONECUT2 AUTS2 RNU7-159P NRG1 ADAMTSL3 RNA5SP360 MIR4497 SYT9 NIPAL2 THRAP3P1 LEXM CDH4 E2F7 RNU6-284P JPH3 GAPDHP50 LINC01148 KAZN SLC14A2 FGD6 ZBTB46 TWIST1 TNR DPP10 LINC02052 PLAA DINOL PGLYRP3 ZNF292 CRPP1 EFCAB3 OLFML1 TBCEL-TECTA CRLF3 PROCR FUT8 LNX1 C8orf87 CNTN6 EEF1A1P36 KCNS3 ARID1A LINC02406 RNU6-1252P SEMA3C TTC7B RERGL CMAHP KAT2B CYP1B1 ATP2B1 PCNX1 ELMOD1 VIPR2 RPH3A GTF2IP2 RNA5SP63 CELF2-DT RN7SKP242 LINC02772 STT3B RPS6KA2 RNU6-667P PMF1-BGLAP FXR1 MVB12B ZCCHC14 ANP32C AJAP1 RPP40P2 ARNT2 RNF213 ZNHIT6 USP38 RPL7AP30
Inflammatory Bowel Disease	388	CCND3P1 RIT2 ERGIC1 ADAM30 IRF4 TRAF3IP2-AS1 HORMAD2 KANK1 LINC01997 INTS11 LINC00604 TUBD1 CRTC3-AS1 LPP KPNA7 FIBP PTPRK DMRT1 OR7E91P RPS21P8 ARPC2 PPIAP9 DPH5 INAVA NCF4 CDC27 SLC17A1 PLAU LINC02745 TRAF3IP2 C10orf55 ADCY7 FOSL2 FGFR1OP2P1 FCGR2A MUC19 P4HA2 SERPINB5 RN7SL72P MIR548H4 GPR65 LINC01882 C17orf67 TMBIM1 SLC22A4 IMPG2 ITLN2 ITLN1 IRF1 PTK2B BANK1 GOT1 C6orf47-AS1 TMEM174 LSP1 IL2RA DUSP22 SLC22A23 PLCG2 FAP TAGAP-AS1 CDYL2 SYN3 HHEX USP15 LINC00698 SMURF1 SHC1 PPP5C LINC02723 CYCSP42 BSN PRKCB LINC00358 AIMP1P2 IL10 CDC14A GPR183 FAM171B RPL23AP12 MAML2 NFATC1 KCNJ4 LINC01620 RASGRP1 BTF3L4P3 FTO GRID2IP SMAD3 LINC01934 SLC2A13 DAP3 CTBP2P2 TYK2 IKZF1 HIF3A DENND1B LINC00484 CTIF RBPJ LRRC8D ZFP36L1 GPBAR1 IRGM UBAC2 GRB7 KLF3-AS1 GYPC PSMG4 IL2 CYP2C18 LINC02128 HDAC7 PLCL1 C5orf66 OSMR PDLIM4 FOS RPL13P2 CDKN2A-DT CCDC26 CD28 CHORDC1P5 OTUD3 RPS6KA4 ZNF300 CAMK2A LINC02341 SPDEF SBNO2 SLAIN2 TWF1 LINC02694 THADA IRF5 ZPBP2 TET2 TNFSF15 HSD17B4 TMEM117 CD6 LINC01989 SP140 DNMT3B NR5A2 FEN1 COMMD7 CPEB4 GPR35 IFNG-AS1 RPL3 RTEL1-TNFRSF6B LINC02132 COX6B1P6 STAT4 MTAP CD40 AQP12B PPBP LRRK2 LACC1 IKZF3 LINC02009 TEX41 ALDH7A1P4 ETS1 SEMA6D OSTF1P1 RNU4ATAC14P RN7SKP211 GCKR LINC00598 IRF6 LINC01082 NUPR1 RELA CCL7 IRF1-AS1 PLA2R1 LPXN NIFKP1 RNA5SP224 GLYAT FLJ31356 FADS2 LTBR TNFRSF14 IL1RL1 KIR3DL2 HLA-DQB1 DUSP29 LINC01475 SLC9A4 BOK-AS1 PTPN2 PDE10A LINC02571 LITAF RIT1 RGS14 CEBPA YBX1P5 LINC01271 CDC37 ACO2 BATF3 RFX6 IL23R MECOM SKAP2 CALM3 MROH3P SUFU SCAMP3 KSR1 EIF2S2P3 LINC02757 RNF186 CARD9 CELSR3 LINC02635 ADCY3 MAGOH3P CRTC3 MAP3K8 PCBP3 BUD13 HLA-DRB1 CIT LINC02773 FERMT1 PLA2G4A DLD LINC01845 BRD2 SMAD7 PARK7 LINC02888 HLA-B TAB2 HLA-DQB3 CUL2 PDGFB IDI1P2 RNASET2 CADPS VSX2 SLC39A11 CXCL5 PITPNM3 SH2B1 DAP ZMIZ1 JAK2 TM9SF4 CD226 RNU4ATAC4P STAT3 FOXP1 CCL20 POFUT1 MAD1L1 OTUD7A PCSK5 RORC IFIH1 IFNGR2 LINC01378 PPIAP34 CDC42SE2 GALC TNFRSF6B NXPE2P1 FCAR SPATA48 EEF1AKMT2 TPTEP2 CTH COL6A1 IL12B DUSP16 POP7 MIR4679-2 SV2C PRXL2B CCDC85B IPMK GRAMD2B LINC02278 NOD2 RN7SL636P C6orf47 PYGO2 TRIB1 NXPE1 NUDT15 TOM1 SPRED2 EEF1A1P27 YWHAQP1 ERRFI1 PSMA2P1 FUT2 LINC01622 RN7SKP144 GNA12 ST7 IL18R1 ANKRD55 SLC7A10 LINC02555 UBE2L3 SNX20 TTC33 ATG16L1 PTGIR HGFAC MST1 MTCO3P1 RPL32P17 GPR18 BACH2 KAT2A ZGPAT ATXN2L GATD3A SERPINB12 LINC02513 LINC02539 BTBD8 NDFIP1 LINC00581 EPO DNAJB12P1 NOTCH1 CDKAL1 MIR3939 RMI2 TSPAN14 CCL2 PUS10 LINC00824 EPHB4 CYLD-AS1 CCND3 IL27 ZNF300P1 HLA-DQA1 RNU6-222P SYNGR1 U2 SATB1-AS1 CAMK2G EMSY THEMIS NHLRC1 MTND4P6 KIF3B IL18RAP SNRPGP8 LINC02357 ZBTB40 CSF2RB RNU6-793P KCP CREM HNRNPA1P41 RPSAP35 PRDM1 ADAD1 DUSP5-DT PNKD PIGCP2 RNU6-925P TAB2-AS1 ZNF831 PLXNA4 LINC01588 CCRL2 CUL1 AMZ1 LINC01075 LINC02791 KIAA1109 EXOC6 PTCD2 MIR3936HG ITGA4

### Construction and analysis of protein–protein interaction network

**Figure 1:**
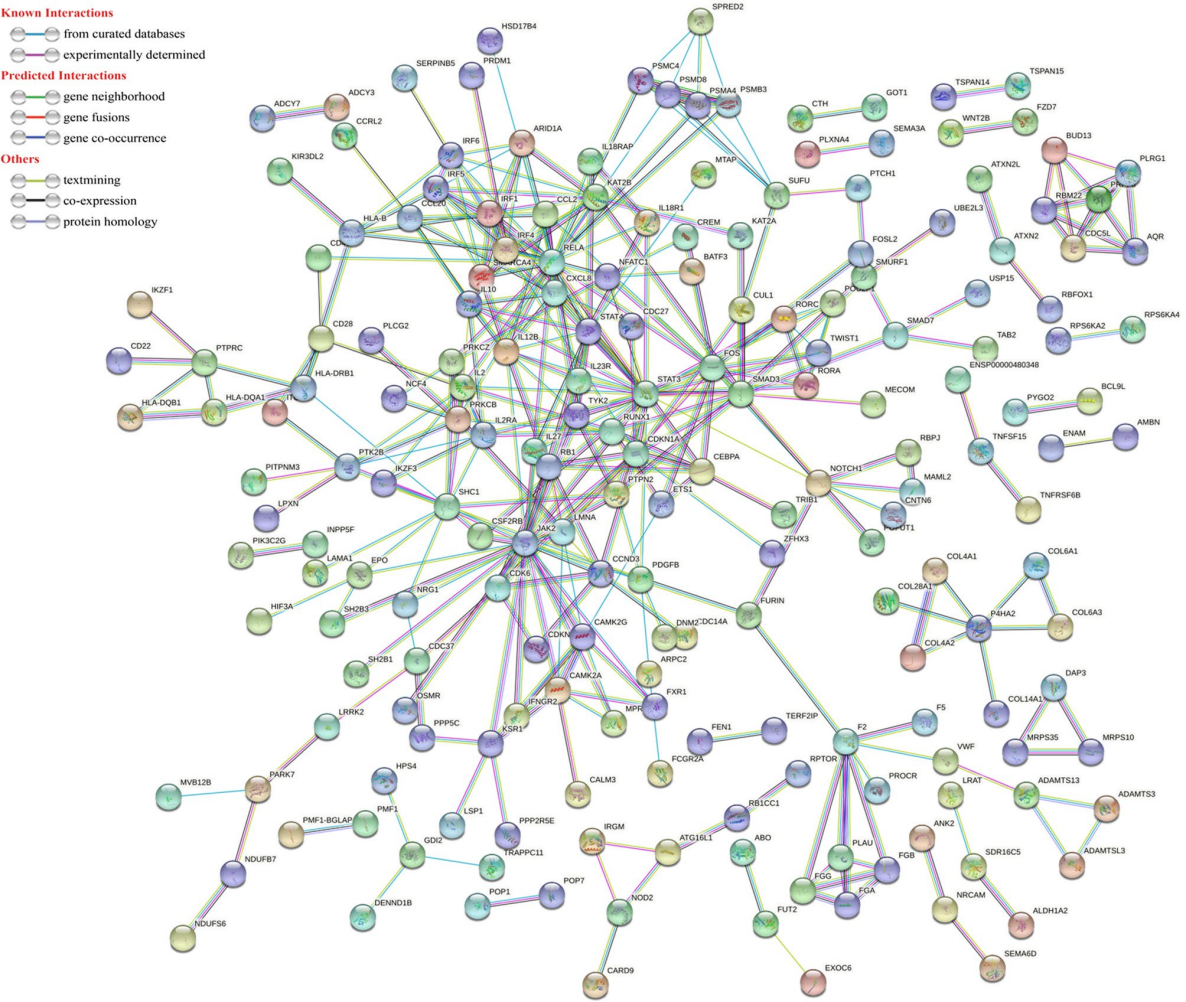
Protein–protein interaction (PPI) network between ischemic stroke and inflammatory bowel disease with minimum required interaction score 0.900, the highest confidence as the cut-off criterion.

**Figure 2:**
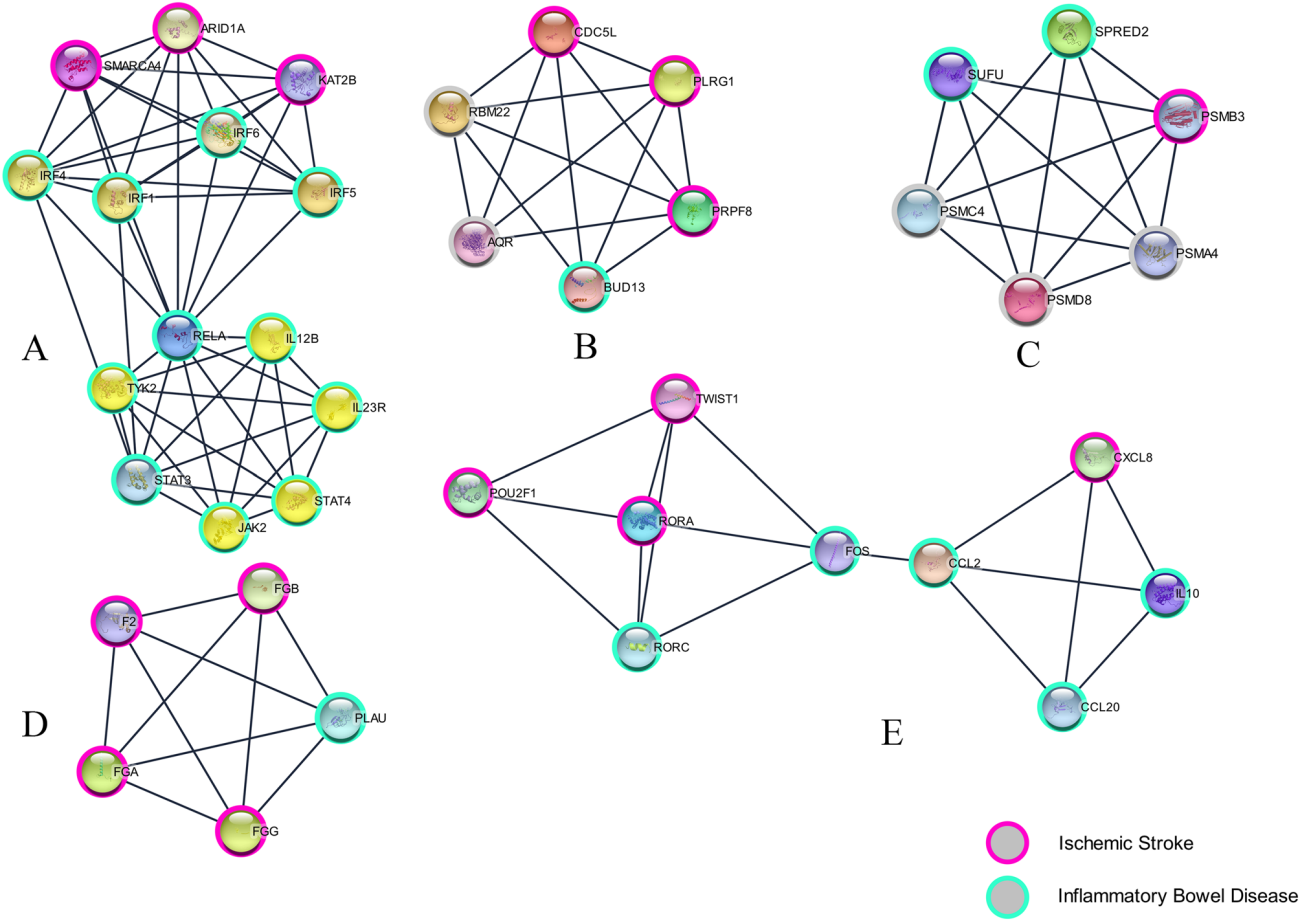
Five significant clusters from the PPI network complex for further analysis using Cytoscape MCODE. (**A**) Cluster 1 consisted of 14 genes primarily associated with Cytokine Signaling in Immune system and Interleukin-23 signaling; (**B**) cluster 2 consisted of 6 genes primarily associated with U2-type spliceosomal complex and Spliceosome; (**C**) cluster 3 consisted of 6 genes primarily associated with Degradation of GLI1 by the proteasome and Proteasome; (**D**) cluster 4 consisted of 5 genes primarily associated with Blood clotting cascade and Fibrinolysis; (**E**) cluster 5 consisted of 9 genes primarily associated with Interleukin-4 and Interleukin-13 signaling and Signaling by Interleukins.

**Figure 3:**
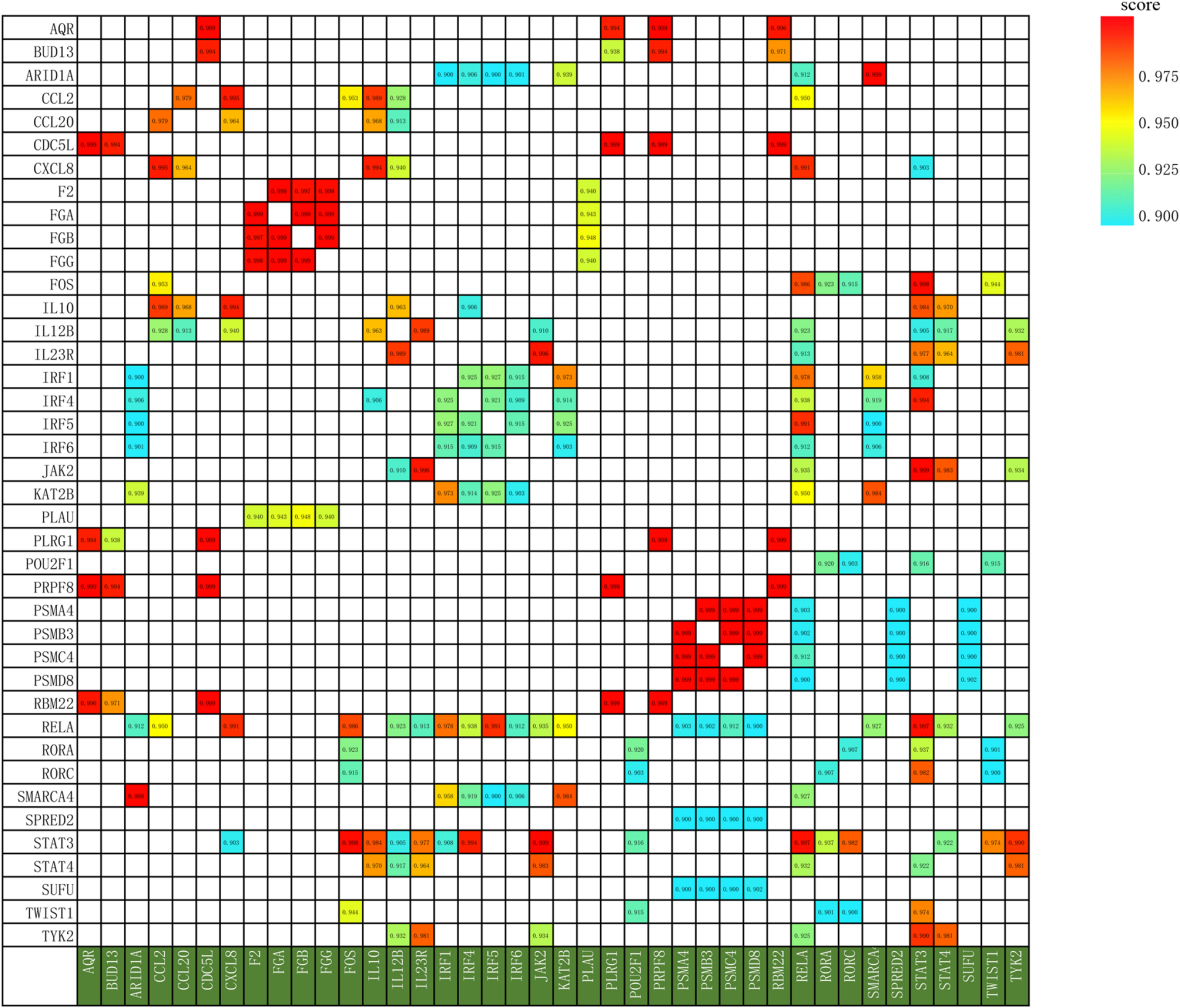
The interactions of the 40 hub node genes.

Using the online web tool STRINGdb and the Cytoscape software platform, a total of 589genes out of the 818 identified, were used to construct a PPI network complex that contained 587 nodes and 366 edges (Fig. 1). According to their degree of importance, we chose five significant clusters from the PPI network complex for further analysis using Cytoscape MCODE. Pathway enrichment analysis showed that cluster 1 (Fig. 2A) consisted of 14 genes primarily associated with Cytokine Signaling in Immune system and Interleukin-23 signaling, cluster 2 (Fig. 2B) consisted of 6 genes primarily associated with U2-type spliceosomal complex and Spliceosome, cluster 3 (Fig. 2C) consisted of 6 genes primarily associated with Degradation of GLI1 by the proteasome and Proteasome, cluster 4 (Fig. 2D) consisted of 5 genes primarily associated with Blood clotting cascade and Fibrinolysis, whereas cluster 5 (Fig. 2E) consisted of 9 genes primarily associated with Interleukin-4 and Interleukin-13 signaling and Signaling by Interleukins. Furthermore,The interactions of the 40 hub node genes are visualized using a heatmap plot (Fig. 3).

### GO and pathway enrichment analysis

**Figure 4:**
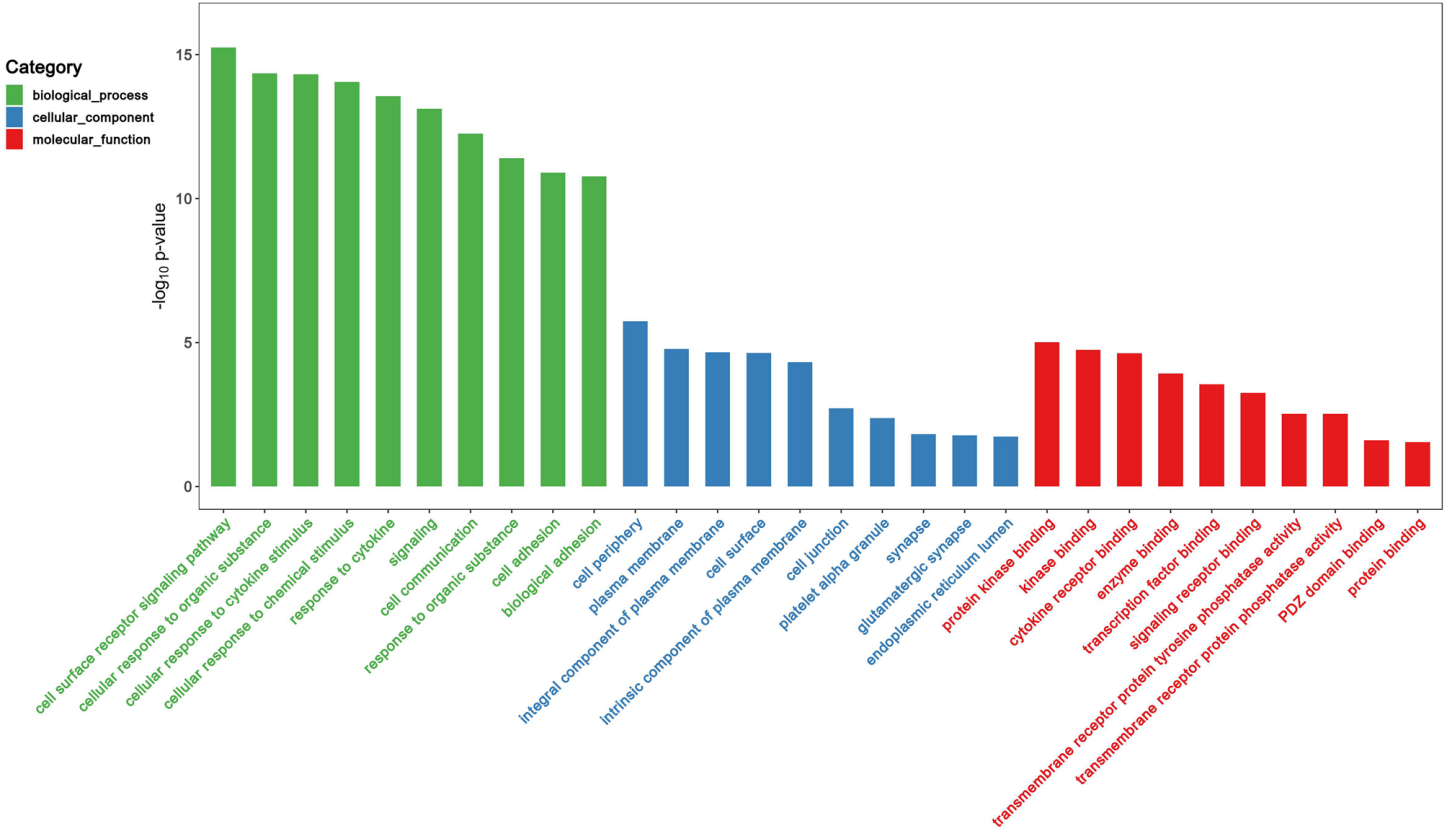
Gene ontology analysis and significant enriched GO terms of genes associated with Ischemic stroke and inflammatory bowel disease. GO analysis classified the DEGs into three groups (i.e., biological process, molecular function and cellular component).

GO and pathway enrichment analyses of these significant genes were performed using multiple online databases. The functional GO terms were classified into three categories: molecular function (MF), biological process (BP), and cellular component (CC). As shown in Fig. 4 and Table 2, in the BP category, the genes were primarily enriched in Cellular response to cytokine stimulus, Cell surface receptor signaling pathway, Response to cytokine, et al. In the MF category, the genes were primarily enriched in Protein binding, Protein kinase binding, Kinase binding, et al. In the CC category, the genes were primarily enriched in Plasma membrane, Cell periphery, Integral component of plasma membrane, et al.. These results show that most of the identified genes were significantly enriched in protein binding, innate immune response, defense response to virus, cytosol, and negative regulation of apoptotic process terms.

**Table 2:** GO and pathway enrichment analyses of significant genes in ischemic strokes and inflammatory bowel disease.

Term	Category	Observed gene count	Strength	False discovery rate [FDR]
Cellular response to cytokine stimulus	BP	92	0.48	7.00E−16
Cell surface receptor signaling pathway	BP	150	0.33	3.29E−15
Response to cytokine	BP	94	0.45	4.04E−15
Signaling	BP	254	0.21	8.51E−14
Cellular response to organic substance	BP	147	0.32	9.24E−14
Cell communication	BP	254	0.2	3.93E−13
Cellular response to chemical stimulus	BP	167	0.28	3.93E−13
Cytokine-mediated signaling pathway	BP	67	0.52	4.15E−13
Signal transduction	BP	238	0.21	4.45E−13
Regulation of response to stimulus	BP	211	0.23	4.45E−13
Protein binding	MF	283	0.13	6.44E−06
Protein kinase binding	MF	47	0.38	0.00026
Kinase binding	MF	50	0.35	0.00045
Binding	MF	433	0.06	0.0005
Signaling receptor binding	MF	83	0.24	0.00091
Cytokine receptor binding	MF	25	0.5	0.0012
Enzyme binding	MF	102	0.18	0.014
Transcription factor binding	MF	41	0.31	0.0172
Immune receptor activity	MF	15	0.58	0.0172
Extracellular matrix structural constituent	MF	14	0.59	0.018
Plasma membrane	CC	220	0.14	7.74E−05
Cell periphery	CC	225	0.14	7.74E−05
Integral component of plasma membrane	CC	88	0.26	8.33E−05
Cell surface	CC	54	0.34	0.00012
Intrinsic component of plasma membrane	CC	90	0.25	0.00012
Side of membrane	CC	36	0.35	0.0046
Cell junction	CC	94	0.18	0.016
Platelet alpha granule	CC	12	0.64	0.016
Receptor complex	CC	27	0.37	0.0192
Endoplasmic reticulum lumen	CC	23	0.4	0.0286

### Pathway enrichment analysis

**Figure 5:**
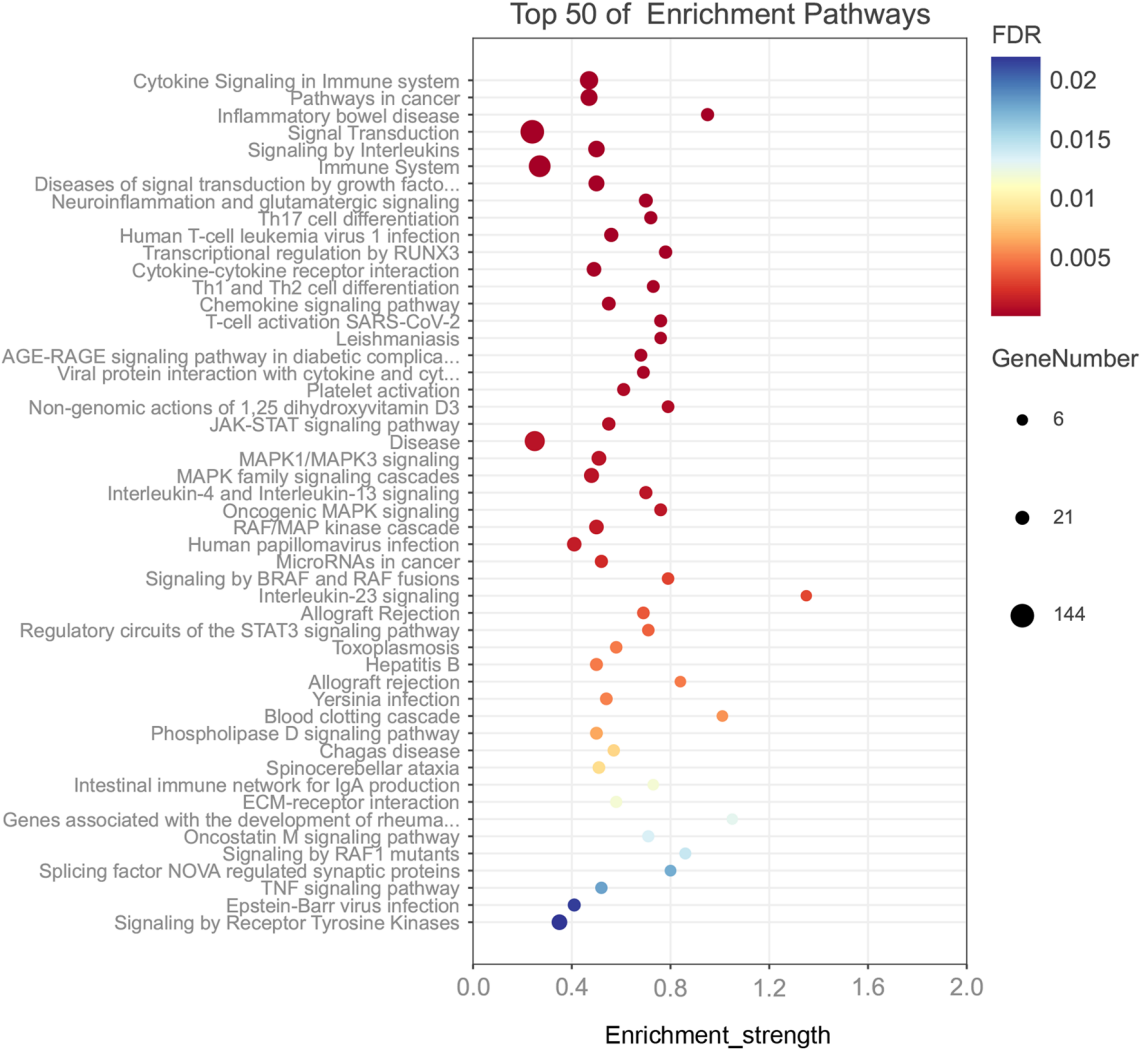
Significantly enriched pathway terms of genes associated with ischemic stroke and inflammatory bowel disease.

Functional and signaling pathway enrichment analyses of these significant genes were conducted using multiple online databases. By KEGG pathway analysis, these genes were mainly enriched in Pathways in cancer, Inflammatory bowel disease, Th17 cell differentiation, Human T-cell leukemia virus 1 infection, Cytokine-cytokine receptor interaction. Furthermore, We used 'Reactome pathways' to analyze these important genes and calculated the false discovery rate using Fisher's Exact, and the results showed that Cytokine Signaling in Immune system, Signal Transduction, Signaling by Interleukins, Immune System, Diseases of signal transduction by growth factor receptors and second messengers, and Transcriptional regulation by RUNX3 were the significant signaling pathways. In addition, we also used "WikiPathways" analysis as a complement to show that these significant genes aremainly enriched in Neuroinflammation and glutamatergic signaling, T-cell activation SARS-CoV-2, Non-genomic actions of 1,25 dihydroxyvitamin D3, Allograft Rejection, and Regulatory circuits of the STAT3 signaling pathway. Together, these results showed that these significant genes had several pathways in common, including those involved in immune system, cytokine signaling in immune system, adaptive immune system (Fig. 5).

### Corroboration of predicted signaling pathways

To further corroborate the key signaling pathways obtained from the above bioinformatic analysis, we used DIA-MS, one of the highly regarded mass spectrometry acquisition techniques in recent years, to identify and quantify proteins in samples from 8 blood samples (4 ischemic stroke samples and 4 normal population samples) (Table 3), 60 down-regulated and 18 up-regulated protein (Tables 4, 5) followed by pathway enrichment analysis with Reactome pathways, and revealed some aspects of over-representation in Complement cascade, Regulation of Complement cascade, Scavenging of heme from plasma, innate immune system and binding and uptake of ligands by scavenger receptors (Table 6).

**Table 3:** Clinical characteristics of 4 ischemic stroke (IS) patients and healthy (HE) (n = 4).

No	Group	Subjects source	Sex	Age	Disease duration (days)	Combined diseases	Crisis	NIHSS score
1	IS	Hospital	Male	63	25	Hypertension, type 2 diabetes	N	7
2	IS	Hospital	Female	55	30	Hypertension, Hyperlipidemia, Chronic Bronchitis	N	10
3	IS	Hospital	Female	55	14	–	N	8
4	IS	Hospital	Female	67	3	Hypertension, Type 2 diabetes, hyperlipidemia	N	8
5	HE	Community	Female	37	–	–	–	–
6	HE	Community	Male	35	–	–	–	–
7	HE	Community	Female	20	–	–	–	–
8	HE	Community	Male	28	–	–	–	–

**Table 4:** 78 differentially expressed proteins (DEPs) were identified from four profile datasets, including 18 up-regulated and 60 down-regulated proteins in ischemic stroke.

Expression	Genes name
Down-regulated	IGLV5-45, IGKV3D-15, IGKV6D-21, IGKV1D-13, QSOX1, APOM, ADH1B, F10, A2M, IGLV2-8, IGHA2, KRT14, APOA1, APOC1, AHSG, TF, PROC, APOB, LCAT, GAPDH, IGKV1-16, SERPINA7, APOA4, CTSD, C8G, MBL2, LAMP1, HSP90B1, JUP, CPN1, DSP, ITIH2, GPX3, CPN2, CRHBP, DPP4, KRT9, IGFALS, AFM, MMP16, PLTP, CFHR1, HGFAC, SPP2, ALCAM, SPARCL1, HABP2, PON3, SPDYA, MEGF8, OAF, GGH, NEO1, CNDP1, FUCA2, COLEC11, COG4, MINPP1, LYVE1, FCGBP
Up-regulated	IGLV10-54, IGKV2-40, IGKJ1, IGHV3-15, IGHV1-24, IGHV3-64D, CP, IGHV2-5, SLC4A1, LRG1, DBH, IGLL1, PROZ, IGLV3-21, CHIT1, PM20D1, OIT3, CFHR4

**Table 5:** The gene ontology (GO) enrichment analysis of differentially expressed proteins/genes in ischemic stroke.

ID	Term	Category	padj	Enrichment score	Genes
**Up-regulated**					
0006958	Complement activation, classical pathway	Biological process	5.69E−08	54.46	IGHV3-15, IGHV1-24, IGHV3-64D, IGHV2-5, IGLL1, IGLV3-21
0050871	Positive regulation of B cell activation	Biological process	1.30E−07	74.82	IGHV3-15, IGHV1-24, IGHV3-64D, IGHV2-5, IGLL1
0006910	Phagocytosis, recognition	Biological process	1.30E−07	73.82	IGHV3-15, IGHV1-24, IGHV3-64D, IGHV2-5, IGLL1
0006911	Phagocytosis, engulfment	Biological process	2.66E−07	62.21	IGHV3-15, IGHV1-24, IGHV3-64D, IGHV2-5, IGLL1
0050853	B cell receptor signaling pathway	Biological process	5.11E−07	53.24	IGHV3-15, IGHV1-24, IGHV3-64D, IGHV2-5, IGLL1
0042742	Defense response to bacterium	Biological process	9.22E−06	29.14	IGHV3-15, IGHV1-24, IGHV3-64D, IGHV2-5, IGLL1
0006955	Immune response	Biological process	0.00017	15.21	IGLV10-54, IGHV2-5, IGLL1, IGLV3-21, CHIT1
0045087	Innate immune response	Biological process	0.00078	10.23	IGHV3-15, IGHV1-24, IGHV3-64D, IGHV2-5, IGLL1
0050900	Leukocyte migration	Biological process	0.00019	25.02	IGHV2-5, DBH, IGLL1, IGLV3-21
0030449	Regulation of complement activation	Biological process	0.00075	34.97	IGHV2-5, IGLV3-21, CFHR4
0005576	Extracellular region	Cellular component	0.00014	5.43	CP, IGHV2-5, LRG1, DBH, , IGLL1, IGLV3-21, CHIT1, CFHR4
0070062	Extracellular exosome	Cellular component	0.01925	3.18	CP, SLC4A1, LRG1, PROZ, IGLV3-21, PM20D1
0005886	Plasma membrane	Cellular component	0.23425	1.45	IGLV10-54, CP, IGHV2-5, SLC4A1, , IGLV3-21
0042571	Immunoglobulin complex, circulating	Cellular component	1.30E−07	81.42	IGHV3-15, IGHV1-24, IGHV3-64D, IGHV2-5, IGLL1
0019814	Immunoglobulin complex	Cellular component	0.00025	52.73	IGLV10-54, , IGLV3-21
1904724	Tertiary granule lumen	Cellular component	0.00612	40.27	LRG1, CHIT1
0035580	Specific granule lumen	Cellular component	0.00748	35.72	LRG1, CHIT1
0005788	Endoplasmic reticulum lumen	Cellular component	0.04555	7.43	CP, PROZ
0043231	Intracellular membrane-bounded organelle	Cellular component	0.17757	2.80	LRG1, DBH
0005783	Endoplasmic reticulum	Cellular component	0.25882	2.13	DBH, IGLL1
0003823	Antigen binding	Molecular function	4.57E−08	63.28	IGHV3-15, IGHV1-24, IGHV3-64D, IGHV2-5, IGLL1, IGLV3-21
0034987	Immunoglobulin receptor binding	Molecular function	1.30E−07	79.10	IGHV3-15, IGHV1-24, IGHV3-64D, IGHV2-5, IGLL1
0005507	Copper ion binding	Molecular function	0.0059	41.79	CP, DBH
0005509	Calcium ion binding	Molecular function	0.15874	3.09	PROZ, OIT3
**Down-regulated**					
0044267	Cellular protein metabolic process	Biological process	4.36E−12	21.42	QSOX1, APOA1, AHSG, TF, PROC, APOB, APOA4, HSP90B1, ITIH2, IGFALS, SPP2, SPARCL1, FUCA2
0043687	Post-translational protein modification	Biological process	4.70E−07	10.38	QSOX1, APOA1, AHSG, TF, PROC, APOB, HSP90B1, ITIH2, SPP2, SPARCL1, FUCA2
0034375	High-density lipoprotein particle remodeling	Biological process	1.02E−09	108.19	APOM, APOA1, APOC1, LCAT, APOA4, PLTP
0033344	Cholesterol efflux	Biological process	4.70E−07	70.56	APOM, APOA1, APOC1, APOB, APOA4
0001523	Retinoid metabolic process	Biological process	4.92E−05	25.76	APOM, ADH1B, APOA1, APOB, APOA4
0043691	Reverse cholesterol transport	Biological process	9.22E−06	76.37	APOM, APOA1, LCAT, APOA4
0042157	Lipoprotein metabolic process	Biological process	1.89E−05	61.82	APOM, APOA1, APOC1, APOA4
0042158	Lipoprotein biosynthetic process	Biological process	1.27E−05	194.74	APOA1, APOB, LCAT
0010873	Positive regulation of cholesterol esterification	Biological process	6.39E−05	108.19	APOA1, APOC1, APOA4
0034371	Chylomicron remodeling	Biological process	6.39E−05	108.19	APOA1, APOB, APOA4
0005576	Extracellular region	Cellular component	2.71E-23	6.89	QSOX1, APOM, F10, A2M, IGLV2-8, IGHA2, APOA1, APOC1, AHSG, TF, PROC, APOB, LCAT, SERPINA7, APOA4, CTSD, C8G, MBL2, HSP90B1, JUP, CPN1, ITIH2, GPX3, CPN2, CRHBP, DPP4, IGFALS, AFM, PLTP, CFHR1, HGFAC, SPP2, SPARCL1, HABP2, PON3, GGH, CNDP1, FUCA2, COLEC11
0070062	Extracellular exosome	Cellular component	5.92E−15	5.13	QSOX1, A2M, IGHA2, KRT14, APOA1, AHSG, TF, APOB, LCAT, GAPDH, SERPINA7, APOA4, CTSD, C8G, LAMP1, HSP90B1, JUP, DSP, ITIH2, GPX3, CPN2, DPP4, KRT9, IGFALS, AFM, ALCAM, PON3, MEGF8, GGH, FUCA2, MINPP1, LYVE1, FCGBP
0005788	Endoplasmic reticulum lumen	Cellular component	2.85E−11	15.25	QSOX1, F10, APOA1, AHSG, TF, PROC, APOB, APOA4, HSP90B1, ITIH2, SPP2, SPARCL1, FUCA2, MINPP1
0062023	Collagen-containing extracellular matrix	Cellular component	4.75E−06	9.41	A2M, APOA1, AHSG, APOA4, CTSD, MBL2, HSP90B1, ITIH2, SPP2, SPARCL1
0034364	High-density lipoprotein particle	Cellular component	5.92E−11	94.67	APOM, APOA1, APOC1, APOB, LCAT, APOA4, PLTP
0034361	Very-low-density lipoprotein particle	Cellular component	2.67E−07	81.14	APOM, APOA1, APOC1, APOB, APOA4
0019814	Immunoglobulin complex	Cellular component	4.92E−05	25.76	IGLV5-45, IGKV3D-15, IGKV6D-21, IGLV2-8,
0042627	Chylomicron	Cellular component	3.26E−06	99.87	APOA1, APOC1, APOB, APOA4
0034362	Low-density lipoprotein particle	Cellular component	0.00017	74.90	APOM, APOA1, APOB
0071682	Endocytic vesicle lumen	Cellular component	0.00023	64.91	APOA1, APOB, HSP90B1
0005543	Phospholipid binding	Molecular function	0.00038	14.89	APOM, F10, APOA1, APOB, APOA4
0004252	Serine-type endopeptidase activity	Molecular function	0.00203	9.72	F10, PROC, DPP4, HGFAC, HABP2
0120020	Cholesterol transfer activity	Molecular function	1.89E−05	61.82	APOA1, APOB, APOA4, PLTP
0031210	Phosphatidylcholine binding	Molecular function	5.97E−05	44.77	APOA1, APOC1, APOA4, PLTP
0004866	Endopeptidase inhibitor activity	Molecular function	0.00016	32.46	A2M, AHSG, ITIH2, SPP2
0060228	Phosphatidylcholine-sterol O-acyltransferase activator activity	Molecular function	2.11E−05	162.28	APOA1, APOC1, APOA4
0086083	Cell adhesive protein binding involved in bundle of His cell-Purkinje myocyte communication	Molecular function	0.0013	129.83	JUP, DSP
0055102	Lipase inhibitor activity	Molecular function	0.00177	108.19	APOA1, APOC1
0008035	High-density lipoprotein particle binding	Molecular function	0.00413	64.91	APOA1, PLTP
0005537	Mannose binding	Molecular function	0.01148	36.06	MBL2, COLEC11

**Table 6:** The significant enriched analysis of differentially expressed proteins (DEPs) in ischemic stroke.

Pathway identifier	Pathway name	#Entities found	#Entities total	Entities ratio	Entities p value	Entities FDR	Submitted entities found
**Down-regulated**							
R-HSA-381426	Regulation of Insulin-like growth factor (IGF) transport and uptake by insulin-like growth factor binding proteins (IGFBPs)	12	127	8.43E−03	3.89E−12	8.53E−10	TF; ITIH2; PROC; AHSG; FUCA2; SPP2; SPARCL1; APOA1; QSOX1; IGFALS; APOB; HSP90B1
R-HSA-8957275	Post-translational protein phosphorylation	11	109	7.23E−03	1.63E−11	1.78E−09	TF; ITIH2; PROC; AHSG; FUCA2; SPP2; SPARCL1; APOA1; QSOX1; APOB; HSP90B1
R-HSA-174824	Plasma lipoprotein assembly, remodeling, and clearance	9	98	6.50E−03	2.84E−09	2.07E−07	APOC1; APOA1; LCAT; APOA4; APOB; A2M; PLTP
R-HSA-8963899	Plasma lipoprotein remodeling	7	56	3.72E−03	2.24E−08	1.21E−06	APOA1; LCAT; APOA4; APOB; PLTP
R-HSA-166658	Complement cascade	9	156	1.04E−02	1.46E−07	6.28E−06	IGLV2-8; COLEC11; C8G; IGLV5-45; IGKV1-16; CFHR1; CPN2; CPN1; MBL2
R-HSA-168249	Innate immune system	23	1334	8.85E−02	2.11E−07	7.59E−06	DSP; COLEC11; JUP; AHSG; FUCA2; GGH; CPN2; CPN1; HSP90B1; IGLV2-8; TF; APOM; C8G; IGLV5-45; IGKV1-16; LAMP1; CFHR1; QSOX1; APOB; CTSD; MBL2
R-HSA-8963898	Plasma lipoprotein assembly	5	30	1.99E−03	6.23E−07	1.93E−05	APOC1; APOA1; APOA4; APOB; A2M
R-HSA-114608	Platelet degranulation	8	141	9.36E−03	8.48E−07	2.29E−05	TF; APOM; LAMP1; AHSG; SPP2; APOA1; QSOX1; A2M
R-HSA-76005	Response to elevated platelet cytosolic Ca2+	8	148	9.82E−03	1.21E−06	2.91E−05	TF; APOM; LAMP1; AHSG; SPP2; APOA1; QSOX1; A2M
R-HSA-2173782	Binding and uptake of ligands by scavenger receptors	8	168	1.11E−02	3.08E−06	6.46E−05	IGLV2-8; COLEC11; IGLV5-45; IGKV1-16; APOA1; APOB; IGHA2; HSP90B1
R-HSA-6798695	Neutrophil degranulation	12	480	3.18E−02	7.66E−06	1.45E−04	DSP; TF; APOM; JUP; LAMP1; AHSG; FUCA2; GGH; QSOX1; CTSD; CPN1
R-HSA-8964058	HDL remodeling	4	24	1.59E−03	8.78E−06	1.47E−04	APOA1; LCAT; PLTP
R-HSA-977606	Regulation of complement cascade	7	139	9.22E−03	9.21E−06	1.47E−04	IGLV2-8; C8G; IGLV5-45; IGKV1-16; CFHR1; CPN2; CPN1
R-HSA-109582	Hemostasis	15	803	5.33E−02	1.65E−05	2.47E−04	F10; AHSG; APOA1; IGLV2-8; TF; PROC; APOM; IGLV5-45; IGKV1-16; LAMP1; SPP2; QSOX1; APOB; A2M; IGHA2
R-HSA-9029569	NR1H3 and NR1H2 regulate gene expression linked to cholesterol transport and efflux	5	66	4.38E−03	2.77E−05	3.88E−04	APOC1; PLTP
R-HSA-8963888	Chylomicron assembly	3	14	9.29E−04	6.01E−05	7.82E−04	APOA1; APOA4; APOB
R-HSA-9024446	NR1H2 and NR1H3-mediated signaling	5	85	5.64E−03	9.09E−05	1.09E−03	APOC1; PLTP
R-HSA-6809371	Formation of the cornified envelope	6	138	9.16E−03	9.25E−05	1.11E−03	DSP; JUP; KRT14; KRT9
R-HSA-8963901	Chylomicron remodeling	3	17	1.13E−03	1.06E−04	1.17E−03	APOA1; APOA4; APOB
R-HSA-3000480	Scavenging by Class A receptors	4	49	3.25E−03	1.38E−04	1.38E−03	COLEC11; APOA1; APOB; HSP90B1
R-HSA-76002	Platelet activation, signaling and aggregation	8	293	1.94E−02	1.56E−04	1.56E−03	TF; APOM; LAMP1; AHSG; SPP2; APOA1; QSOX1; A2M
R-HSA-2168880	Scavenging of heme from plasma	5	106	7.03E−03	2.52E−04	2.27E−03	IGLV2-8; IGLV5-45; IGKV1-16; APOA1; IGHA2
R-HSA-166786	Creation of C4 and C2 activators	5	111	7.37E−03	3.11E−04	2.80E−03	IGLV2-8; COLEC11; IGLV5-45; IGKV1-16; MBL2
R-HSA-140837	Intrinsic pathway of fibrin clot formation	3	26	1.73E−03	3.68E−04	3.31E−03	PROC; F10; A2M
R-HSA-166663	Initial triggering of complement	5	120	7.96E−03	4.42E−04	3.54E−03	IGLV2-8; COLEC11; IGLV5-45; IGKV1-16; MBL2
R-HSA-975634	Retinoid metabolism and transport	4	79	5.24E−03	8.29E−04	6.64E−03	APOM; APOA1; APOA4; APOB
R-HSA-8866423	VLDL assembly	2	9	5.97E−04	1.07E−03	7.46E−03	APOC1; APOB
R-HSA-159763	Transport of gamma-carboxylated protein precursors from the endoplasmic reticulum to the Golgi apparatus	2	9	5.97E−04	1.07E−03	7.46E−03	PROC; F10
R-HSA-6805567	Keratinization	6	226	1.50E−02	1.24E−03	7.87E−03	DSP; JUP; KRT14; KRT9
R-HSA-6806667	Metabolism of fat-soluble vitamins	4	89	5.91E−03	1.28E−03	7.87E−03	APOM; APOA1; APOA4; APOB
R-HSA-8964046	VLDL clearance	2	10	6.64E−04	1.31E−03	7.87E−03	APOC1; APOB
R-HSA-159782	Removal of aminoterminal propeptides from gamma-carboxylated proteins	2	10	6.64E−04	1.31E−03	7.87E−03	PROC; F10
R-HSA-8964043	Plasma lipoprotein clearance	3	42	2.79E−03	1.46E−03	8.77E−03	APOC1; APOA1; APOB
R-HSA-140877	Formation of fibrin clot (clotting cascade)	3	43	2.85E−03	1.56E−03	9.37E−03	PROC; F10; A2M
R-HSA-202733	Cell surface interactions at the vascular wall	6	257	1.71E−02	2.36E−03	1.42E−02	IGLV2-8; PROC; IGLV5-45; IGKV1-16; APOB; IGHA2
R-HSA-166662	Lectin pathway of complement activation	2	15	9.95E−04	2.90E−03	1.45E−02	COLEC11; MBL2
R-HSA-159740	Gamma-carboxylation of protein precursors	2	15	9.95E−04	2.90E−03	1.45E−02	PROC; F10
R-HSA-381183	ATF6 (ATF6-alpha) activates chaperone genes	2	15	9.95E−04	2.90E−03	1.45E−02	HSP90B1
R-HSA-159854	Gamma-carboxylation, transport, and amino-terminal cleavage of proteins	2	16	1.06E−03	3.29E−03	1.64E−02	PROC; F10
R-HSA-381033	ATF6 (ATF6-alpha) activates chaperones	2	17	1.13E−03	3.70E−03	1.85E−02	HSP90B1
R-HSA-8963896	HDL assembly	2	18	1.19E−03	4.13E−03	2.07E−02	APOA1; A2M
R-HSA-9006931	Signaling by nuclear receptors	7	387	2.57E−02	4.25E−03	2.13E−02	APOC1; PLTP; CTSD
R-HSA-3000471	Scavenging by Class B receptors	2	21	1.39E−03	5.57E−03	2.23E−02	APOA1; APOB
R-HSA-140875	Common pathway of fibrin clot formation	2	25	1.66E−03	7.79E−03	3.12E−02	PROC; F10
R-HSA-392499	Metabolism of proteins	20	2207	1.46E−01	8.85E−03	3.54E−02	ITIH2; F10; AHSG; COG4; FUCA2; APOA1; APOA4; HSP90B1; DPP4; TF; PROC; SPP2; SPARCL1; QSOX1; IGFALS; APOB; CTSD; GAPDH
R-HSA-168256	Immune system	23	2684	1.78E−01	9.29E−03	3.71E−02	DSP; COLEC11; JUP; AHSG; FUCA2; GGH; CPN2; CPN1; HSP90B1; IGLV2-8; TF; APOM; C8G; IGLV5-45; IGKV1-16; LAMP1; CFHR1; QSOX1; APOB; CTSD; MBL2
R-HSA-8963889	Assembly of active LPL and LIPC lipase complexes	2	30	1.99E−03	1.10E−02	4.41E−02	APOA4
R-HSA-5653656	Vesicle-mediated transport	10	827	5.49E−02	1.11E−02	4.45E−02	IGLV2-8; COLEC11; TF; IGLV5-45; IGKV1-16; COG4; APOA1; APOB; IGHA2; HSP90B1
R-HSA-977225	Amyloid fiber formation	3	89	5.91E−03	1.17E−02	4.67E−02	TF; APOA1; APOA4
R-HSA-382551	Transport of small molecules	11	966	6.41E−02	1.19E−02	4.76E−02	TF; APOC1; APOA1; LCAT; APOA4; APOB; A2M; NEO1; PLTP
R-HSA-2187338	Visual phototransduction	4	169	1.12E−02	1.22E−02	4.88E−02	APOM; APOA1; APOA4; APOB
**Up-regulated**							
R-HSA-977606	Regulation of complement cascade	5	135	1.16E−02	2.87E−06	1.55E−04	IGHV2-5; IGLV3-21; IGLL1; CFHR4; IGLV10-54
R-HSA-166658	Complement cascade	5	146	1.26E−02	4.20E−06	1.55E−04	IGHV2-5; IGLV3-21; IGLL1; CFHR4; IGLV10-54
R-HSA-173623	Classical antibody-mediated complement activation	4	95	8.20E−03	1.97E−05	2.70E−04	IGHV2-5; IGLV3-21; IGLL1; IGLV10-54
R-HSA-2168880	Scavenging of heme from plasma	4	99	8.54E−03	2.31E−05	2.70E−04	IGHV2-5; IGLV3-21; IGLL1; IGLV10-54
R-HSA-2029481	FCGR activation	4	101	8.72E−03	2.50E−05	2.70E−04	IGHV2-5; IGLV3-21; IGLL1; IGLV10-54
R-HSA-2730905	Role of LAT2/NTAL/LAB on calcium mobilization	4	102	8.80E−03	2.60E−05	2.70E−04	IGHV2-5; IGLV3-21; IGLL1; IGLV10-54
R-HSA-166786	Creation of C4 and C2 activators	4	103	8.89E−03	2.70E−05	2.70E−04	IGHV2-5; IGLV3-21; IGLL1; IGLV10-54
R-HSA-166663	Initial triggering of complement	4	111	9.58E−03	3.61E−05	2.83E−04	IGHV2-5; IGLV3-21; IGLL1; IGLV10-54
R-HSA-2029485	Role of phospholipids in phagocytosis	4	114	9.84E−03	4.00E−05	2.83E−04	IGHV2-5; IGLV3-21; IGLL1; IGLV10-54
R-HSA-2871809	FCERI mediated Ca + 2 mobilization	4	117	1.01E−02	4.42E−05	2.83E−04	IGHV2-5; IGLV3-21; IGLL1; IGLV10-54
R-HSA-2871796	FCERI mediated MAPK activation	4	119	1.03E−02	4.72E−05	2.83E−04	IGHV2-5; IGLV3-21; IGLL1; IGLV10-54
R-HSA-202733	Cell surface interactions at the vascular wall	5	246	2.12E−02	5.12E−05	3.07E−04	IGHV2-5; IGLV3-21; IGLL1; IGLV10-54
R-HSA-9664323	FCGR3A-mediated IL10 synthesis	4	128	1.10E−02	6.26E−05	3.13E−04	IGHV2-5; IGLV3-21; IGLL1; IGLV10-54
R-HSA-2173782	Binding and uptake of ligands by scavenger receptors	4	129	1.11E−02	6.45E−05	3.22E−04	IGHV2-5; IGLV3-21; IGLL1; IGLV10-54
R-HSA-9664417	Leishmania phagocytosis	4	149	1.29E−02	1.12E−04	4.49E−04	IGHV2-5; IGLV3-21; IGLL1; IGLV10-54
R-HSA-9664407	Parasite infection	4	149	1.29E−02	1.12E−04	4.49E−04	IGHV2-5; IGLV3-21; IGLL1; IGLV10-54
R-HSA-9664422	FCGR3A-mediated phagocytosis	4	149	1.29E−02	1.12E−04	4.49E−04	IGHV2-5; IGLV3-21; IGLL1; IGLV10-54
R-HSA-2029482	Regulation of actin dynamics for phagocytic cup formation	4	150	1.29E−02	1.15E−04	4.61E−04	IGHV2-5; IGLV3-21; IGLL1; IGLV10-54
R-HSA-2871837	FCERI mediated NF-kB activation	4	167	1.44E−02	1.74E−04	5.21E−04	IGHV2-5; IGLV3-21; IGLL1; IGLV10-54
R-HSA-2029480	Fcgamma receptor (FCGR) dependent phagocytosis	4	175	1.51E−02	2.08E−04	6.23E−04	IGHV2-5; IGLV3-21; IGLL1; IGLV10-54
R-HSA-5690714	CD22 mediated BCR regulation	3	70	6.04E−03	2.33E−04	6.98E−04	IGHV2-5; IGLV3-21; IGLL1
R-HSA-2454202	Fc epsilon receptor (FCERI) signaling	4	218	1.88E−02	4.77E−04	1.43E−03	IGHV2-5; IGLV3-21; IGLL1; IGLV10-54
R-HSA-983695	Antigen activates B cell receptor (BCR) leading to generation of second messengers	3	95	8.20E−03	5.66E−04	1.70E−03	IGHV2-5; IGLV3-21; IGLL1
R-HSA-9664433	Leishmania parasite growth and survival	4	259	2.23E−02	9.07E−04	1.81E−03	IGHV2-5; IGLV3-21; IGLL1; IGLV10-54
R-HSA-9662851	Anti-inflammatory response favouring Leishmania parasite infection	4	259	2.23E−02	9.07E−04	1.81E−03	IGHV2-5; IGLV3-21; IGLL1; IGLV10-54
R-HSA-198933	Immunoregulatory interactions between a lymphoid and a non-lymphoid cell	4	297	2.56E−02	1.50E−03	3.01E−03	IGHV2-5; IGLV3-21; IGLL1; IGLV10-54
R-HSA-9673163	Oleoyl-phe metabolism	1	1	8.63E−05	1.72E−03	3.45E−03	PM20D1
R-HSA-5619050	Defective SLC4A1 causes hereditary spherocytosis type 4 (HSP4), distal renal tubular acidosis (dRTA) and dRTA with hemolytic anemia (dRTA-HA)	1	1	8.63E−05	1.72E−03	3.45E−03	SLC4A1
R-HSA-9658195	Leishmania infection	4	345	2.98E−02	2.60E−03	5.19E−03	IGHV2-5; IGLV3-21; IGLL1; IGLV10-54
R-HSA-168249	Innate immune system	7	1191	1.03E−01	2.79E−03	5.59E−03	CHIT1; LRG1; IGHV2-5; IGLV3-21; IGLL1; CFHR4; IGLV10-54
R-HSA-983705	Signaling by the B cell receptor (BCR)	3	176	1.52E−02	3.29E−03	6.58E−03	IGHV2-5; IGLV3-21; IGLL1
R-HSA-5619049	Defective SLC40A1 causes hemochromatosis 4 (HFE4) (macrophages)	1	2	1.73E−04	3.45E−03	6.89E−03	CP
R-HSA-5619060	Defective CP causes aceruloplasminemia (ACERULOP)	1	2	1.73E−04	3.45E−03	6.89E−03	CP
R-HSA-109582	Hemostasis	5	726	6.26E−02	6.76E−03	1.35E−02	IGHV2-5; IGLV3-21; IGLL1; IGLV10-54
R-HSA-209905	Catecholamine biosynthesis	1	4	3.45E−04	6.88E−03	1.37E−02	DBH
R-HSA-166187	Mitochondrial uncoupling	1	6	5.18E−04	1.03E−02	1.37E−02	PM20D1
R-HSA-5619102	SLC transporter disorders	2	103	8.89E−03	1.35E−02	1.37E−02	SLC4A1; CP
R-HSA-1247673	Erythrocytes take up oxygen and release carbon dioxide	1	8	6.90E−04	1.37E−02	1.37E−02	SLC4A1
R-HSA-159763	Transport of gamma-carboxylated protein precursors from the endoplasmic reticulum to the Golgi apparatus	1	9	7.77E−04	1.54E−02	1.54E−02	PROZ
R-HSA-425381	Bicarbonate transporters	1	10	8.63E−04	1.71E−02	1.71E−02	SLC4A1
R-HSA-159782	Removal of aminoterminal propeptides from gamma-carboxylated proteins	1	10	8.63E−04	1.71E−02	1.71E−02	PROZ
R-HSA-159740	Gamma-carboxylation of protein precursors	1	10	8.63E−04	1.71E−02	1.71E−02	PROZ
R-HSA-2534343	Interaction with cumulus cells and the zona pellucida	1	11	9.49E−04	1.88E−02	1.88E−02	CHIT1
R-HSA-159854	Gamma-carboxylation, transport, and amino-terminal cleavage of proteins	1	11	9.49E−04	1.88E−02	1.88E−02	PROZ
R-HSA-1480926	O2/CO2 exchange in erythrocytes	1	12	1.04E−03	2.05E−02	2.05E−02	SLC4A1
R-HSA-1237044	Erythrocytes take up carbon dioxide and release oxygen	1	12	1.04E−03	2.05E−02	2.05E−02	SLC4A1
R-HSA-189085	Digestion of dietary carbohydrate	1	12	1.04E−03	2.05E−02	2.05E−02	CHIT1
R-HSA-209776	Metabolism of amine-derived hormones	1	18	1.55E−03	3.06E−02	3.06E−02	DBH
R-HSA-5619115	Disorders of transmembrane transporters	2	181	1.56E−02	3.85E−02	3.85E−02	SLC4A1; CP
R-HSA-5653656	Vesicle-mediated transport	4	761	6.57E−02	3.86E−02	3.86E−02	IGHV2-5; IGLV3-21; IGLL1; IGLV10-54
R-HSA-8935690	Digestion	1	24	2.07E−03	4.06E−02	4.06E−02	CHIT1
R-HSA-1187000	Fertilization	1	26	2.24E−03	4.39E−02	4.39E−02	CHIT1
R-HSA-425410	Metal ion SLC transporters	1	26	2.24E−03	4.39E−02	4.39E−02	CP
R-HSA-8963743	Digestion and absorption	1	29	2.50E−03	4.89E−02	4.89E−02	CHIT1

## Discussion

For this exploratory bioinformatics analysis, potential PPI networks between ischemic stroke and inflammatory bowel disease were predicted using the open catalog of human GWAS studies. Although this predicted PPI network requires careful interpretation and preclinical and clinical validation, it shows new evidence that will contribute to new studies on the relationship between ischemic stroke and inflammatory bowel disease. In the following we propose different hypothetical biological scenarios depending on our PPI network, by which inflammatory bowel disease can increase the risk of ischemic stroke.

It has long been known that cardioembolism, large artery atherosclerosis, and small vessel occlusion are three main pathophysiological mechanisms in patients with IS^18^. Moreover, cerebral ischemia is responsible for producing various immune cell mediators, which can exacerbate ischemic brain injury. CNS inflammation induced by ischemic conditions plays an essential role in stroke pathophysiology and exacerbates infarct formation at the injury site^19,20^.

As previously mentioned, in our study, the five most significant clusters from the PPI network were identified by integrated bioinformatics analysis. These clusters contained 40 hub genes. The first one consisted of 14 genes (*KAT2B, IRF4, IL23R, SMARCA4, IRF1, IRF5, IRF6, RELA, IL12B, STAT4, STAT3, JAK2, TYK2, ARID1A*) (Fig. 4B) whose biological functions could be associated with Cytokine Signaling in Immune system, Interleukin-23 signaling, Signaling by Interleukins, Th17 cell differentiation, and JAK-STAT signaling pathway. By analyzing the first cluster, we found that various immune cell mediators influence the various processes of atherosclerosis mainly by affecting inflammation, with cytokines and interleukin-related signaling having a particularly significant impact. IRF5 expression was significantly increased in peripheral blood mononuclear cells (PBMCs) and colonic inflammatory tissue in patients with IBD and was significantly associated with IBD activity^21^. The activated IRF5 increased macrophage infiltration and lipid accumulation, with concomitant reductions in smooth muscle cells and collagen content, weakening plaque stability and promoting the formation of atherosclerotic plaques ^22^. KAT2B upregulation promotes acetylation of HMGB1, promotes pro-inflammatory cell polarization and macrophage recruitment, and promotes atherosclerotic progression ^23^. Dysregulation of KAT2B may lead to inhibition of IL-10, a key anti-inflammatory cytokine, disrupting innate and adaptive inflammatory responses and promoting the development of inflammatory bowel disease^24^. In peripheral inflammation, interferon regulatory factor-5 (IRF5) and IRF4 regulate M1 and M2 activation of macrophages, respectively, leading to a greater risk of ischemia in the elderly brain^25^. Traditional T cells coordinate the inflammatory response in atherosclerosis and their function is altered by the lipoprotein milieu and complement activity. Hypercholesterolemic states are associated with upregulation of DAF expression in circulating T cells and increased levels of nuclear factor kappa B (NF-kB) and interferon regulatory factor 4 (IRF4) ^26^. The inhibition of IRF5 expression and promotion of IRF4 expression in lamina propria monocytes induces macrophage polarization to the M2 phenotype, which improves the inflammatory response in inflammatory bowel disease^27^, and increased IRF4 expression results in enhanced M2 activation, reduced pro-inflammatory response, and improved stroke prognosis, whereas downregulation of IRF4 results in increased IRF5 expression, enhanced M1 activation, increased pro-inflammatory response, and functional poorer recovery^28^. The inflammatory response, immune response, and cytokine-cytokine receptor interactions may play an important role in the progression of atherosclerosis. Co-operative inflammatory signaling by TLRs, STAT/IRF, and IFNs leads to M1 polarization of macrophages, which contributes to the development of atherosclerosis^29^. In response to TNF-α stimulation, Ataxin-10 promoted the cytoplasmic localization of IRF-1, thereby inhibiting VCAM-1 transcription ^30^. IL23R-dependent gamma-delta T cells can release IL-17 and GM-CSF, which together induce inflammation and necrosis in macrophages. Il-23R + gamma-delta T cells locally promote early lesion formation in the aortic root and contribute to the expansion of the necrotic core, a hallmark of vulnerable atherosclerotic lesions ^31^. Interferon regulatory factor 1 (IRF-1) contributes to the pathological phenotype of VSMCs during atherosclerosis by increasing CCL19 transcription, whereas silencing IRF-1 inhibits angiotensin proliferation and migration and downregulates CCL19 expression, thereby suppressing atherosclerosis ^32^. RelA-driven escalation of pro-inflammatory gene responses in intestinal epithelial cells exacerbates inflammatory cell infiltration and colonic lesions, driving inflammation associated with inflammatory bowel disease (IBD) ^33^. Upregulation of RELA /p65 inhibits Hif-1α, thereby suppressing neuroinflammatory activity after ischemic stroke, and attenuating brain injury caused by cerebral ischemia^34^. SMARCA4/BAF190A may promote neural stem cell self-renewal/proliferation by enhancing Notch-dependent proliferative signaling while insensitizing neural stem cells to SHH-dependent differentiation cues. Inflammation of endothelial cells induces inflammatory cytokine production and monocyte adhesion, which are key events in initiating atherosclerosis. aberrant activation of STAT3 has been shown to contribute to the development and progression of atherosclerosis, and signal transducer and activator of transcription 3 (STAT3) have key roles in endothelial cell dysfunction, macrophage polarization, inflammation, and immunity during atherosclerosis^35^. Reduced phosphorylation of signal transducer and activator of transcription 3 (STAT3) and nuclear levels of STAT3 and interferon-regulated transcription factor-1 (IRF1), which in turn inhibits the STAT3/IRF1 pathway in vascular endothelial cells, produce anti-atherosclerotic effects on TNF-α-induced inflammation^36^. As the primary pathological basis of ischemic stroke disease, its pathogenesis has been shown to involve an imbalance in anti-inflammatory/pro-inflammatory processes. IL-23R can enhance its antigen-presenting capacity through an autocrine pathway, allowing it to infiltrate the lesion site, promoting its secretion of large amounts of inflammatory factors, and upregulating pro-inflammatory DCs and macrophages. The IL-23 binds to IL-23R on the surface of target cells through Janus kinase 2/signal transducer and transcriptional activator channels to deliver signals involved in chronic inflammatory and autoimmune diseases^37^. Among the nine IRFs, at least three (IRF-1, IRF-5, and IRF-8) are involved in pro-inflammatory M1 commitment, while IRF-3 and IRF-4 control M2 polarization^38^. IRF6 is a potential causative gene for ischemic stroke^39^. STAT4 deletion reduces perivascular and visceral AT inflammation, which may reduce atheromatous plaque formation through direct or indirect effects^40^. The mRNA levels of IL-23 and IL-23R were significantly higher in carotid plaques compared to non-atherosclerotic vessels. The coalition showed co-localization with plaque macrophages. IL-23 accentuated tumor necrosis factor release in monocytes from patients with carotid atherosclerosis, and high plasma levels of IL-23 may increase mortality. IL-23 is associated with disease progression in patients with carotid atherosclerosis and may be involved in IL-17-related mechanisms^41^. Inhibition of TNF-α-induced phosphorylation of IkappaB-α and nuclear translocation of NF-kappaB P65 (RELA) attenuated TNFα-induced expression of ICAM-1, VCAM-1 and E-selectin, which had a significant benefit in delaying the progression of inflammatory diseases, including atherosclerosis^42^. ARID1A, a subunit of the SWItch/Sucrose Non-Fermentable (SWI/SNF) chromatin remodeling complex, is localized to promoters and enhancers to influence transcription to affect the progression of atherosclerosis^43^. 

Spliceosome and mRNA Splicing-Major Pathway are the most relevant signaling pathways in the second cluster, and this cluster consists of BUD13, RBM22, CDC5L, PRF8, PLRG1, and AQR. The main molecular events after ischemic stroke are translation-related, which may be caused by subsequent signaling pathway-related effects caused by upregulated cell membrane ribosomal and spliceosomal complex proteins^44^. BUD13, a subunit of the retention and splicing complex, may significantly increase the risk of ischemic stroke by affecting APOA1 leading to elevated TG and VLDL^45,46^. PLRG1 is a core component of the complex encoding the cell division cycle 5-like (CDC5L) complex. The CDC5L complex is part of the spliceosome, and both are highly conserved spliceosomal proteins across species. They are both shown to be part of the secondary spliceosomal protein complex and their encoded proteins play a critical role in alternative splice site selection. The interaction between CDC5L and PLRG1 is important for pre-mRNA splicing and essential for human pre-mRNA splicing^47^. Transcript variants of this gene with alternative splicing have been observed, encoding multiple isoforms^48^. CDC5L is significantly associated with the diagnosis of atrial fibrillation and ischemic stroke^49^. Knockdown of AQR in HepG2 facilitates glucose uptake, decreases PCK2 expression levels, increases GSK-3β phosphorylation, and restores insulin sensitivity^50^. The Innate immune genes are differentially expressed during either adaptation to hypoxia or recovery from H/R stress. Prolyl hydroxylase EGL-9, a known regulator of adaptation to hypoxia and innate immune response, inhibits the rapid recovery from H/R stress by activating AQR activity in O2-sensing neurons^51^. PPRF8 (pre-mRNA processing factor 8), a core component of the spliceosome, is an important mediator of hypoxia-induced mitosis^52^, and a high-fat diet upregulates the expression of genes related to the spliceosome^53^, increasing the risk of ischemic stroke. Adjusting the diet may enhance functional recovery after stroke by modulating insulin resistance and normalizing glucose metabolism^54^.

The third cluster contains six genes, SPRED2, PSMA4, PSMC4, PSMB3, SUFU, PSMD8, which are mainly involved in Degradation of GLI1, GLI2and GLI3 by the proteasome and Proteasome signaling pathways. Atherosclerosis is the primary pathological basis of ischemic stroke. In addition to its intensive inflammatory character, atherosclerosis also has features of enhanced proliferation, apoptosis, and oxidative stress. Besides playing an essential role in the degradation of dysfunctional and oxidatively damaged proteins, the ubiquitin–proteasome system (UPS), the major intracellular degradation system in eukaryotic cells, is involved in many processes that influence the progression of atherosclerotic disease^55^, which in turn affects the outcome of ischemic brain injury. SPRED2 is a negative regulator of the extracellular signal-regulated kinase (ERK) pathway and is important for cell proliferation, neuronal differentiation, plasticity, and survival. The decrease in SPRED-2 after injury may be related to the activation of the ERK pathway, and SPRED-2 signaling may play a role in the cell proliferation phase of neural repair in the zebrafish brain after injury^56^. SPRED2 affects neurological recovery after stroke by reducing cell migration through the ERK/c-Fos/MMPs pathway^57^. By binding to Gli, Sufu regulates the expression of target genes downstream of the Hedgehog (Hh) signaling pathway, and Sufu alternates between "open" and "closed" conformations, with the "closed" form of Sufu stabilized by Gli binding and inhibited by Hh processing. In contrast, the "open" state of Sufu is facilitated by the separation of Gli and Hh signaling^58^, which in turn affects neural regeneration in the brain. Down-regulation of three subunits of the proteasome (PSMA2, PSMA3, and PSMA4) genes to regulate cellular functions against oxidative stress, which has the effect of reducing atherosclerosis^59^. Specific proteasome inhibitor PR957 inhibited LMP7 and significantly attenuated histological damage to the cerebral white matter and the cognitive function in ischemic stroke by suppressing the inflammatory response^60^.

The fourth cluster is mainly related to thrombosis and coagulation signaling pathways, containing five genes PLAU, FGG, F2, FGB, and FGA. The three genes FGG, FGB, and FGA encode fibrinogen, which is a separate risk factor for ischemic stroke, like hypertension and diabetes mellitus^61^. In addition, single nucleotide polymorphisms (SNPs) in FGG and FGA mediate an increase in D-dimers^62^, which are significantly associated with stroke progression^63^. Fibrinogen (Fg) levels decrease in the colon of inflammatory bowel disease (IBD), which plays a crucial role in the pathogenesis of IBD by regulating vascular permeability (VP) through activation of AKT and subsequent microfilament depolymerization^64^. The level of intraplatelet PLAU is closely related to the production of intraplatelet plasma proteins and the secondary degradation of α-granulin, which affects fibrinolysis^65^. Moreover, PLAU may play a role in the treatment of ulcerative colitis^66^. Binding of recombinant PLAU (uPA) or endogenous uPA to uPAR induces membrane recruitment and activation of β1 integrins via low-density lipoprotein receptor-associated protein-1 (LRP1), causing activation of the Rho family small GTPase Rac1 and Rac1-induced axonal regeneration, which promotes neuronal growth during development and functional improvement after ischemic injury^67,68^. The F2 gene encodes thrombospondin (also known as coagulation factor II), which is proteolytically cleaved in multiple steps to form activated serine protease thrombin. The activated thrombin plays an essential role in thrombosis and hemostasis by converting fibrinogen to fibrin during clot formation, stimulating platelet aggregation, and animating other coagulation factors.

Interestingly, the fifth cluster also mainly involves the interleukin signaling pathway, and the interaction of FOS and CCL2 genes in this cluster may play a bridging role. FOS is a potential target gene for Crohn's disease (CD)^69^, where downregulation of FOS significantly enhances oxidative stress levels, accelerates neuronal apoptosis, and inhibits mitochondrial function, increasing the risk of ischemic stroke^70^. CCL2 is intimately associated with unstable atherosclerosis as upregulated based on inflammatory stimuli. Inhibition of CCL2 expression attenuates the polarization of pro-inflammatory microglia and inhibits the release of inflammatory cytokines such as TNF-α, IFN-γ, IL-1β, IL-6, IL-12, IL-17, and IL-23 while acting as a neuroprotective agent^71,72^. Overexpression of CCL2 is closely associated with colonic inflammation in inflammatory bowel disease^73^. IL-23 and IL-17 induce platelet-activating factor receptor (PAF-R) expression on activated T cells, which is invoked by PAF binding to PAF-R. PAF-R is co-expressed with IL-17 and similarly regulated with the Th17 markers IL-17A, IL-17F, IL-22, and RORC^74^. Through STAT3, IL-6 and IL-21 promote the expression of RORA and RORC genes encoding RORα and RORγt, respectively, RORγ(t) is an essential regulator of Th17 cell differentiation. These two RORs then drive the expression of IL-17A/F and IL-22. Inverse RORγ(t) agonists cause a rebalancing of the Th17/Treg ratio in favor of anti-inflammatory Tregs^75^. RORA is not only overexpressed in both Crohn's disease (CD) and ulcerative colitis (UC)^76^, but is also involved in cholesterol metabolism^77^. RORgammat( +) Treg cells in patients with inflammatory bowel disease are progressively enlarged and have pro-inflammatory properties^78^. Endothelial-to-mesenchymal transition (EndMT) plays a significant role in atherosclerosis, and Twist-1^79^, a prime regulator of T helper cells adapted to chronic inflammatory metabolism, and its activation induces this process^80^. Many cytokines and chemokines are involved in the process of atherosclerotic plaque formation, either accelerating the progression of atherosclerotic plaque or slowing down this process. Plasma levels of IL-8/CXCL8 in patients with acute ischemic stroke tend to increase over time^81^. Serum CXCL8 levels are associated with infarct size and functional outcome in patients with ischemic stroke^82^. And IL10 causes IL-1beta and TNF-alpha to be down-regulated, which may effectively reduce inflammation in atherosclerotic plaques^83^. Chemokine CXCL12, on the other hand, exerts a protective role against atherosclerosis^84^. Furthermore, atherosclerosis is an inflammatory disease of the vessel wall characterized by the activation of the innate immune system with macrophages as the main actors and the adaptive immune system with Th1 as the primary factor. In advanced atherosclerosis, a defect in outflow cells drives disease progression, and the expansion of regulatory T cells (TREG) ameliorates the role of outflow cells. Treg cells secrete interleukin-13 (IL-13), which stimulates macrophage production of IL-10. Endocrine paracrine signaling of IL-10 induces Vav1 in macrophages and promotes phagocytosis by apoptotic cells^85^. In addition, Treg cells promote macrophage efflux and improve inflammatory conditions by enhancing the transcytotic signaling pathway of apoptotic cell internalization^86^.

Finally, the results of bioinformatics analysis of proteins significantly differentially expressed in four ischemic stroke patients and four healthy subjects suggested that the Complement cascade related signaling pathways, Innate Immune System, and hemostasis related signaling pathways were the most significantly associated signaling pathways in ischemic stroke. Although this is not entirely consistent with the signaling pathways associated with ischemic stroke and inflammatory bowel disease obtained from previous analyses, it also suggests that the immune system, signal transduction, and hemostasis-related pathways are key signaling pathways in ischemic stroke. There are two main limitations of this study. First, due to the lag in the inclusion of proteins and protein interactions in bioinformatics such as string, many newly discovered proteins are not available with corresponding matching data, and the results may be further updated with the addition of data from subsequent related studies. However, because of the universality of this study, which included more than one million people and involved multiple populations, the results are of good value. Second, we did not perform further protein validation, but since this study used the DIA-MS technique, which has high accuracy, so further validation was carried out in the next phase. Our study may help us understand the relationship between ischemic stroke and inflammatory bowel disease and, with the help of advances in proteomics such as DIA-MS, help further understand the molecules and cytokines involved in inflammatory signaling in the gut-brain axis, which is essential to our understanding of neuroimmune communication and help develop immunomodulatory therapies for ischemic stroke.

## Conclusion

In conclusion, by performing bioinformatic analysis of two datasets downloaded from NHGRI-GWAS, including ischemic stroke and inflammatory bowel disease, we constructed a protein interaction network containing 589 genes, identified a core protein-related interaction network composed of 40 hub genes, and determined that cytokine and interleukin-related signaling pathways, spliceosome, ubiquitin–proteasome system (UPS), thrombotic and anticoagulant signaling pathways are closely associated with ischemic stroke and inflammatory bowel disease. And these findings could significantly improve our understanding of the potential molecularly linked events in IS and IBD. Furthermore, these candidate genes and pathways could serve as predictive and therapeutic targets for both states (Supplementary Information S1).

## Supplementary Information

Supplementary Information.

## Data Availability

The datasets used and/or analysed during the current study available from the corresponding author on reasonable request.

## Abbreviations

IS: Ischemic stroke
IBD: Inflammatory bowel disease
PPI: Protein–protein interaction
UPS: Ubiquitin–proteasome system
DIA: Data independent acquisition
GO: Gene ontology
DEPs: Differentially expressed proteins
