# Supplementary material for: Exploring the hub mechanisms of ischemic stroke based on protein-protein interaction networks related to ischemic stroke and inflammatory bowel disease
Source: Sci Rep. 2023 Jan 31;13:1741. doi: 10.1038/s41598-023-27459-w (PMC9887582; doi:10.1038/s41598-023-27459-w)
Supplement: Supplementary file 1 — Supplementary Information. [file 41598_2023_27459_MOESM1_ESM.docx]

URL: <https://www.iprox.cn/page/SSV024.html;url=1653655800547jvCy>

Password: mkQU

Valid: 90days
